# Comparison of vitrified and unvitrified Eocene woody tissues by TMAH thermochemolysis – implications for the early stages of the formation of vitrinite

**DOI:** 10.1186/1467-4866-7-9

**Published:** 2006-10-10

**Authors:** Paul E Kaelin, William W Huggett, Ken B Anderson

**Affiliations:** 1 Department of Geology, Southern Illinois University Carbondale, Carbondale, IL, 62901, USA

## Abstract

Samples of vitrified and unvitrified Eocene woody plant tissues collected from the Fossil Forest site, Geodetic Hills, Axel Heiberg Island, have been characterized by TMAH thermochemolysis. All samples are gymnosperm-derived, are of very low maturity and all share the same post-depositional geologic history. Differences in the distributions of products observed from vitrified and unvitrified samples suggest that vitrification of woody tissue is associated with modification of the lignin C3 side chain, following loss of all or most of the carbohydrate present in the precursor woody tissues. The key driver of vitrification appears to be physical compression of the tissue following biological removal of cellulosic materials.

## Background

Vitrinite is the geologic product resulting from diagenetic alteration of lignocellulosic (woody) plant tissues. It is a major component of most coals and accounts for a significant fraction of sedimentary organic matter (SOM) on a global scale, especially SOM derived from terrestrial biomass. Vitrification, used in this context to refer to the conversion of lignocellulosic tissues to vitrinite, is a key process affecting the long-term burial of woody plant tissues. In general, both hemi-cellulose and cellulose are greatly reduced or completely lost, and lignin-derived structures are selectively enriched [1,2] during early diagenesis of woody plant tissues. Subsequent vitrification involves further chemical and physical modification of this carbohydrate-depleted product, including loss of recognizable tissue structure and conversion into a semi-glassy material.

Numerous authors have investigated the structural characteristics of vitrinite, especially in relation to understanding the overall structural characteristics of coals, and to some extent much of the voluminous work that has been reported on coal maturation is relevant to the structural changes that occur in vitrinite as it progresses through the geologic column. The structural changes that occur during the initial stages of vitrification; that is, the earliest conversion of recognizably woody tissue into glassy vitrified material are, however, much less thoroughly investigated. This step is critical in that it converts relatively labile, bio-available carbon into relatively resistant, largely bio-unavailable carbon, Hence this process is a key step in the long-term burial and sequestration of terrestrial organic carbon.

In the present study, woody and vitrified plant tissues collected from Eocene deposits in the Canadian Arctic have been characterized by thermochemolysis with tetramethylammonium hydroxide (TMAH) to investigate structural changes associated with the initial vitrification of woody plant tissues. Samples were collected from the Fossil Forest site, located in the Geodetic Hills region of Axel Hieberg Island [3]. This deposit is well known for the exceptional preservation of the plant fossils found there. In this case, both "woody" and vitrified tissues were collected, and in the case of vitrified materials, samples of both aerial (branch) and root tissues were recovered. Obst et al [2] have previously described similar samples collected from this and other sites located in the Canadian Arctic.

**Figure 1:**
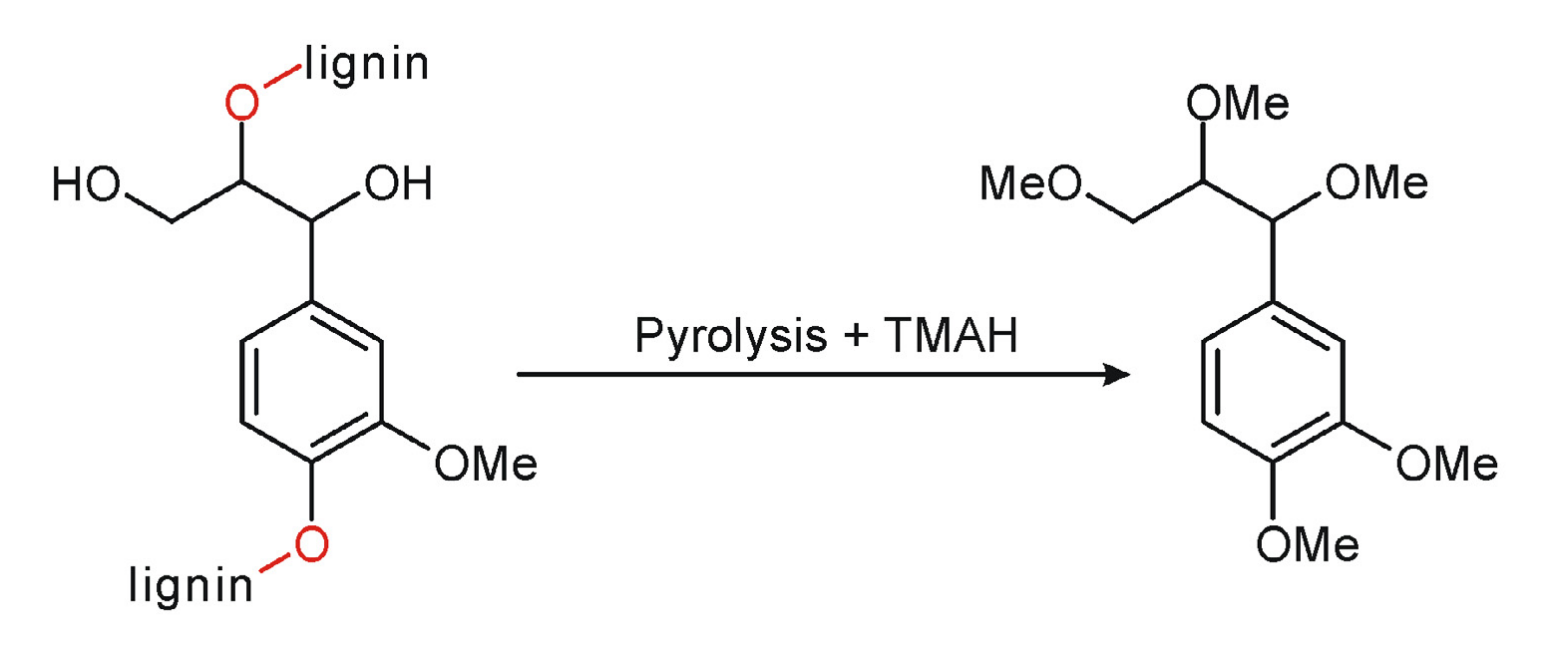
Generic model for thermochemolysis of β-O-4 aliphatic-aryl ether bonds in guaiacyl lignin. Additional reactions also occur, resulting in the formation of numerous related and derived C9–C14 products. Modified after Filley et al [18]. See also [7,13,15,19,] for additional details.

TMAH Thermochemolysis is a thermally assisted hydrolysis technique that is widely used for analysis of humic and other macromolecular materials [4,5] including lignin [6-10]. The technique has been applied to analysis of both recent [6-10] and fossiliferous [11-14] lignocellulosic samples and the mechanism of action appears to be reasonably well understood [14]. As applied to lignin, the process simultaneously hydrolyzes β-O-4 aliphatic-aryl ether bonds, resulting in depolymerization of the macromolecular structure into individual monomers (e.g., guaiacyl and sryingyl units) and simultaneously methylates acidic and phenolic oxygenated structures. See Figure 1. Thermochemolysis does not hydrolyze β-β, β-5 or other non-ether types of inter (lignin) monomer linkages [15,16]. Thermochemolysis can be done offline, but is also amenable to online systems and can be coupled directly with Gas Chromatography-Mass Spectrometry (GC-MS), which facilitates identification of the products that are generated [17].

This technique has major advantages over conventional pyrolysis for analysis of lignin-related/lignin-derived materials [6,13,15,16]. Conventional pyrolysis cleaves lignin polymers into smaller representative compounds, but also results in significant secondary reactions including condensation and repolymerization reactions. The volatile products released consist of monomeric and oligomeric phenols of varying degrees of polarity. Those products with a higher degree of polarity are prone to poor chromatographic behavior, resulting in biased data favoring less polar analytes [6,13,15,16]. In contrast, TMAH thermochemolysis is less prone to interference from secondary reactions and methylation of acidic/phenolic functional groups results in improved chromatographic and overall analytical performance.

## Experimental

### Samples

**Figure 2:**
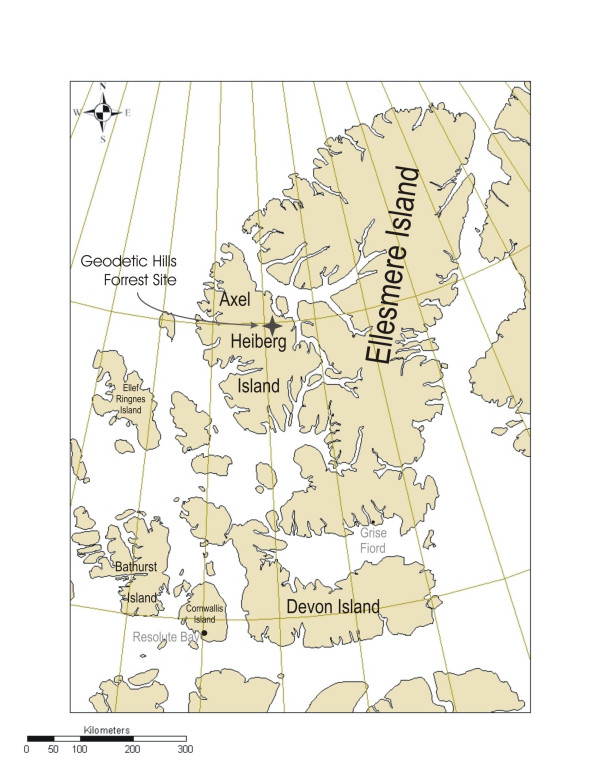
Location of Fossil Forest site, Geodetic Hills, Axel Heiberg Island, Canadian Arctic Archipelago. (Additional details are given in references 2,3,20 and 21).

Samples used in this investigation were collected from the Fossil Forrest site, Geodetic Hills, Axel Heiberg Island, Canadian Arctic (Figure 2.) The site, and the fossiliferous materials located there have been described in detail elsewhere [2,3,20,21]. Deposits in this formation are known to be Middle-Late Eocene and have never been deeply buried [21,22]. All samples characterized were collected from accessible exposures in the upper part of the section. Vitrified samples were collected from organic-rich (coaly) layers. Unvitrified samples were collected from unconsolidated sandy interseam sediments vertically separated from the coaly layers by <~3 m. All samples are of very low maturity, R_o _= 0.11, (slightly outside the range of reflectance values (0.14–0.47) reported by Goodarzi and coworkers [23] for similar samples), consistent with estimates of ~100–200 m maximum depth of burial [21].

The samples used for the present study are largely comparable with those described by Obst and coworkers [2] except that based on the descriptions given, all of the Geodetic Hills samples characterized by those workers were vitrified. Unvitrified samples characterized by Obst et al were collected separately at other sites and hence may have been subject to different depositional or geologic histories. Nevertheless, much of the data reported by Obst and coworkers [2] for samples collected at the Geodetic Hills site are relevant to the samples collected for the present study and readers are referred to this work [2] for an excellent discussion of the general characteristics of woody fossils collected from this site.

**Figure 3:**
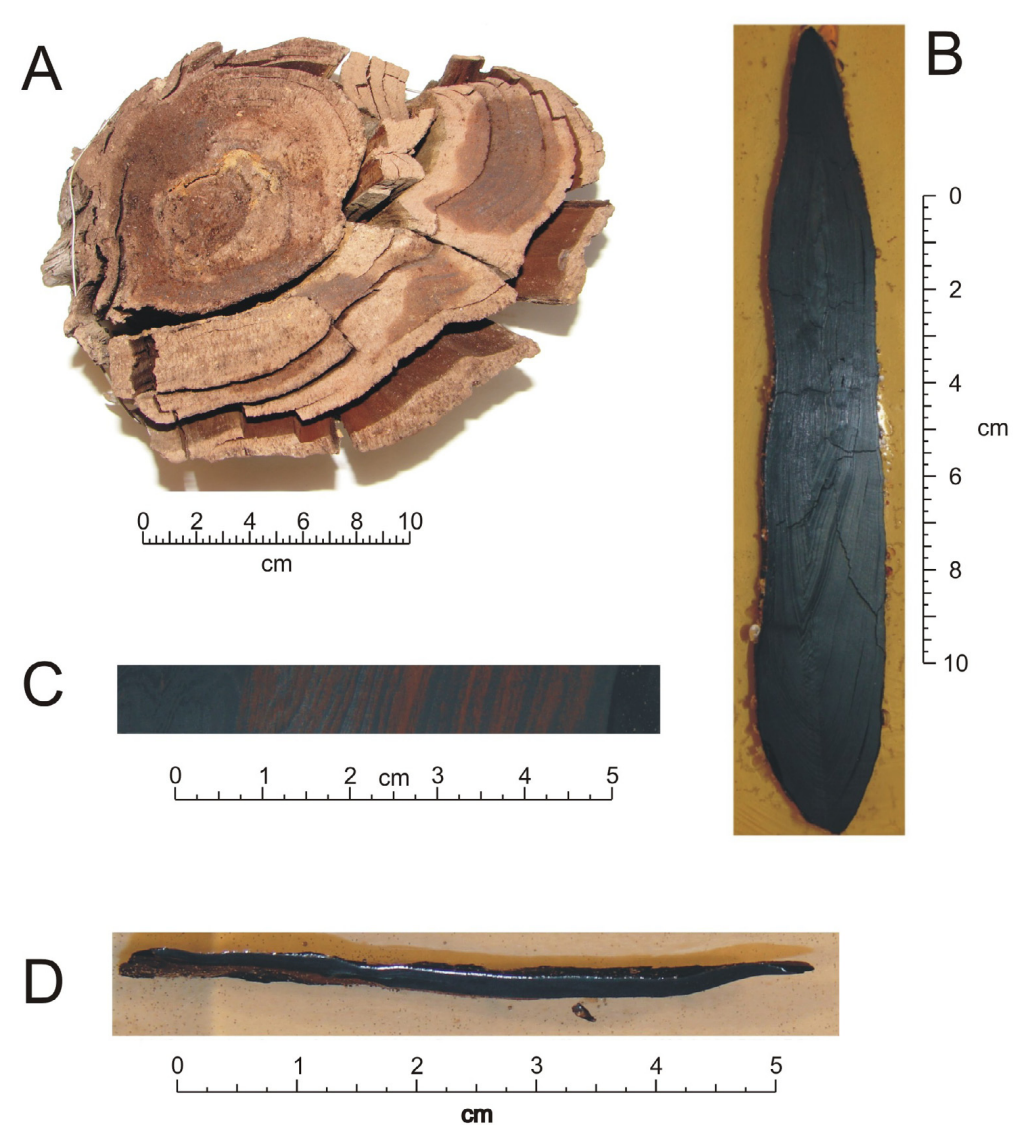
Samples characterized in the present study. A. Unvitrified woody tissue. (Differences in color are due to incomplete drying). B and C. Vitrified aerial (branch) tissue illustrated in polished cross section (B) and polished parallel with the bedding plane (C). D. Vitrified root tissue illustrated in polished cross section.

The samples used in the present study are illustrated in Figure 3. Samples described as woody, or unvitrified (Figure 3A), are visually well preserved uncompressed tissues. Growth rings and other well preserved macroscopic structures are clearly apparent and these samples retain a brownish woody color. Two vitrified samples (branch and root-derived) are also described. These samples are compressed parallel to the bedding plane and retain fewer macroscopically observable characteristics than the uncompressed sample, although in some cases compressed ring structures are still visible in polished cross section. Branch and root tissues are differentiated on the basis of recognizable preserved macroscopic characteristics (general shape, character and form of branching etc) and compression ratio, defined as width parallel to the bedding plane divided by mean thickness parallel to the bedding plane. These samples are black in color, break with a conchoidal fracture, and are harder and denser than the unvitrified samples. Aerial (branch) tissue (Figure 3B and 3C) is identified on the basis of its lower compression ratio (~6:1) and asymmetry. Branches often exhibit asymmetric compression due to the differences in wood density of tissues under compression and tension; lower and upper parts of the branch respectively. In polished cross section (Figure 3B) this sample appears completely vitrified, but when polished parallel with the bedding plane (Figure 3C), some less altered layers become apparent. Care was taken in sub-sampling this sample for thermochemolysis to minimize inclusion of unvitrified materials.

Vitrified root tissue (Figure 3D) is characterized by a high compression ratio (>20:1 in these samples) resulting from the lower initial density of the tissue. Roots do not have to support their own weight and are intended to take up maximum moisture; hence, they tend to be softer and less dense overall than aerial woody plant tissues and consequently tend to compress to a greater degree during compaction following burial.

### Thermochemolysis

In this investigation, 500 ± 200 μg of sample was loaded into a Pyrex capillary tube (0.8–1.1 × 10–12 mm.). Two millimeters of solid TMAH pentahydrate 97% (Aldrich) was then packed into the capillary tube, then two μL of methanol was added to the tube. The mixture was allowed to stand undisturbed for twenty minutes at room temperature, then pyrolyzed (480°C for 10 sec) using a CDS AS-2500 pyrolyzer. Products were separated using an Agilent 6890 GC equipped with a 30 m VB-5 (0.25 mm id, 0.25 μm film thickness,) capillary GC column and identified by mass spectrometry (Agilent 5973 MSD).

## Results and discussion

The primary objective of the present study is comparison of the structural characteristics of vitrified and unvitrified lignocellulosic fossils with essentially identical geologic histories. The availability of samples of both vitrified and unvitrified materials from the same deposit ensures that all samples have the same post-burial geologic history and, given the very low thermal maturity of the samples, facilitates investigation of the structural changes that occur in lignocellulosic tissues during the earliest stages of vitrification. However, since the precursor material (woody tissue) from which the samples used in this study are derived is naturally inhomogeneous, and since the sub-samples used for analysis are small (typically 0.5 ± 0.2 mg), the question of how representative individual analyses are, is significant. Therefore, in order to ensure the validity and significance of differences in data obtained from individual samples, considerable effort was spent to ensure the reproducibility of the data generated.

### Reproducibility

**Figure 4:**
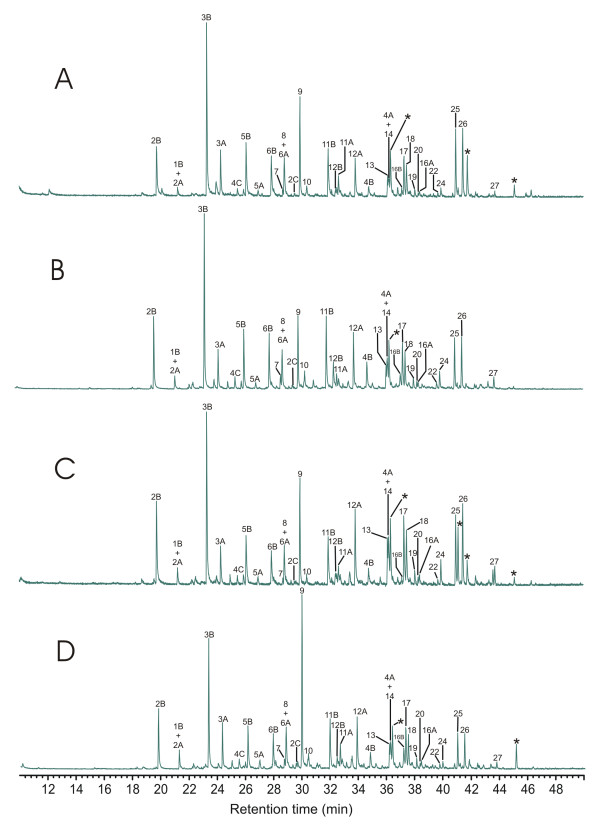
Thermochemolysis-GC-MS data for four discrete analyses of vitrified root tissue, illustrating run-run variability/reproducibility. Data were obtained from multiple analyses of a single sample. * = Contaminant (primarily septum bleed).

**Figure 5:**
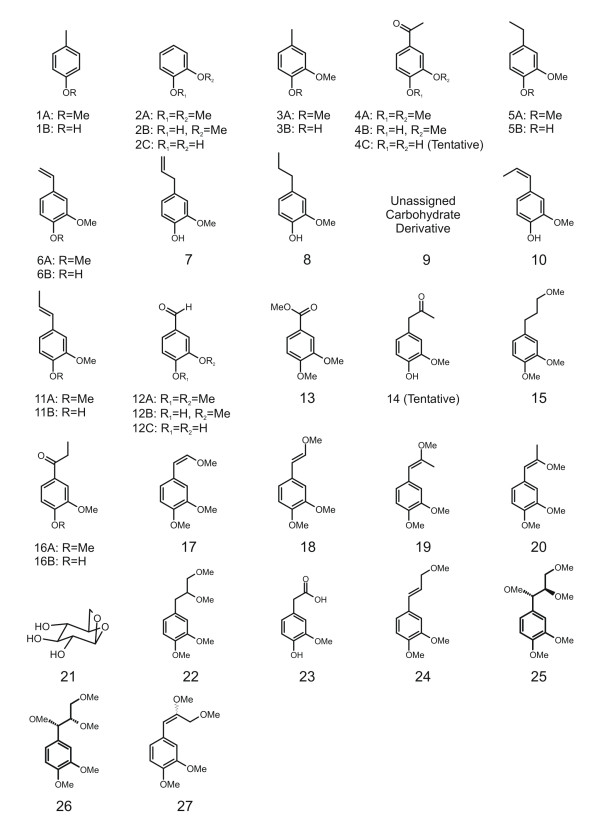
Structure key for assigned eluants identified in Figures 4 and 6.

Preliminary investigations of these samples using experimental techniques that have been shown to give reproducible data for other types of samples [e.g. [24]] yielded inconsistent results. This is due in part to intra-sample inhomogeneity, but also results from inconsistency in the degree of methylation of phenolic products under some experimental conditions. A number of experimental procedures were tested to resolve this problem and the experimental procedure described above was eventually developed. Using this procedure, consistent results are obtained in most cases. Run-run variability is still observed in some analyses, probably due primarily to sample inhomogeneity, but sufficiently reproducible data are typically obtained. Overall run-run reproducibility/variability is illustrated in Figure 4, which shows a stacked plot of 4 different runs of vitrified root tissue. Quantitative treatment of these data, combined with data from analysis of other samples, demonstrating the limits within which differences between discrete samples can be considered significant are discussed further below. Structures for assigned eluants are illustrated in Figure 5. Supplemental data supporting the assignments reported is given in Additional File 1.

### Comparison of vitrified and unvitrified tissues

All of the major products observed in these data have been previously reported by other workers in TMAH thermochemolysis products derived from lignin or lignin-related and derived samples [6,7,14-16,19]. Based on the distribution of products observed it is apparent that these samples are composed of guaiacyl lignin, implying a gymnospermous origin, consistent with paleobotanical evidence of the flora contributing to the deposits found at this site [20] and anatomical investigations of related samples by Obst et al [2]. Only compounds 9 and 21 are attributable to derivation from carbohydrates. This is consistent with a high degree of loss of cellulose and hemi-cellulose during diagenesis [2] and derivation of the resulting vitrinite largely or near exclusively from residual lignin.

**Figure 6:**
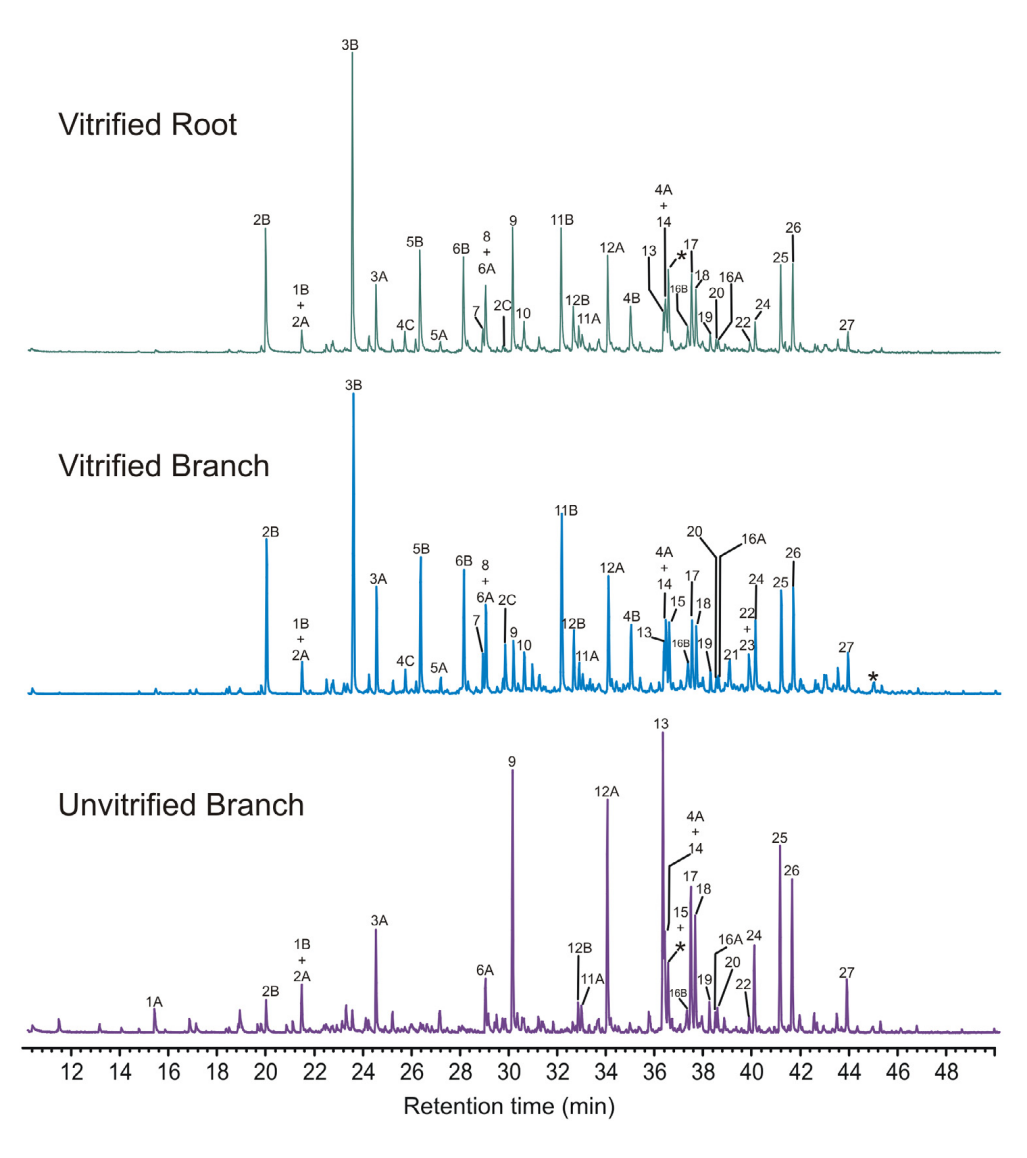
Comparison of thermochemolysis-GC-MS data for vitrified root and branch tissue and unvitrified branch tissue, illustrating differences in the distributions of the products observed.

**Figure 7:**
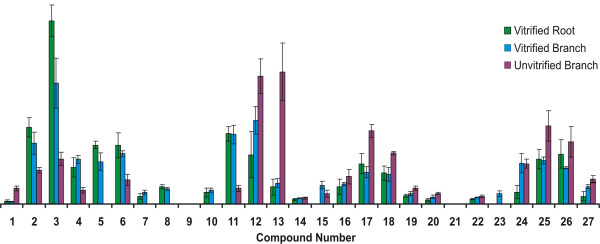
Comparison of distributions of lignin-derived analytes from thermochemolysis of vitrified root and branch and unvitrified branch tissues. Bars are mean values, plotted as a percentage of the total abundance of all lignin-derived products. Error bars represent one standard deviation (n = 4).

Py-GC-MS data from vitrified (root and branch) and unvitrified (branch) tissues are illustrated in Figure 6. Supplemental data supporting the assignments given are available in Additional File 2. Quantitative results comparing data from multiple analyses are shown in Figure 7. To facilitate comparison of multiple samples, data in Figure 7 are normalized to the total abundance of lignin-derived products, and the abundances of products differing only in degree of methylation are summed (e.g. 3 = 3A+3B). Numbering of analytes identified in Figures 6 and 7 is the same as used in Figure 4 and illustrated in Figure 5.

Differences in the data derived from the two vitrified samples are subtle and are generally within the range of intra-sample variation, as described above, indicating that the structural characteristics of these samples are at least grossly similar. Differences between the data derived from vitrified and unvitrified tissues, however, are more significant. The pyrolysate of the unvitrified wood is dominated by compounds 12,13,17,18,25 and 26. Studies by Kuroda and coworkers [15,16,19], and others [14] with model compounds have shown that all of these products are directly attributable to hydrolysis of β-O-4 structures in guaiacyl lignin. These products are also observed in the pyrolysates of the vitrified tissues, however, in the vitrified samples, these products (Numbers 12, 13, 17, 18, 25 and 26) are less abundant than products (Numbers 2, 3, 5, 6 and 11) which have been reported in studies of model compounds to be derived from lignin with modified or alternate inter-monomer linkages (such, for example β-5 and β-β lignin structures) [15,16].

In their report [2], Obst and coworkers reported similar observations based on analytical pyrolysis results (i.e. without thermochemolysis), but in that case they attributed the differences in the observed distributions of products to differences in the age of the samples. The results in this study, in which there is no significant difference in the either the age or geologic history of the samples, indicate that the observed differences reflect structural changes associated with the vitrification process. That is, changes associated with conversion of recognizably woody tissues to vitrinite.

Various authors have attempted to apply parameters such as acid: aldehyde ratio (13:12) and other proxies in TMAH studies of lignin and lignin degradation, as a measure of the degree of degradation or modification of the initial lignin structure. However, in the opinion of the present authors, the usefulness of these parameters in TMAH thermochemolysis studies of lignin is open to question. It is well known that treatment of aldehydes and even alcohols with hydroxide bases at elevated temperatures results in Cannizarro-type reactions [25-27] which have the potential to affect these proxies. Klingberg et al., [28] for example have reported that the numeric value of the acid:aldehyde ratio measured for a consistent (modern) milled wood lignin sample varied by a factor of more than two depending on the amount of TMAH used and other experimental variables.

Consideration of the data illustrated in Figure 6, indicates that the degree of methylation of some products varies considerably between vitrified and unvitrified samples. This suggests differences in the degree of interaction of TMAH with the substrate during thermochemolysis. These differences are probably the result of differences in the porosity/permeability of the samples, and hence differences in the relative rates of evolution of volatile products during pyrolysis, and effective contact between TMAH and the substrate during pyrolysis. If so, the relative abundances of the compounds used to calculate these proxies (12,13 and others) are likely to be considerably dependant on the physical properties of the samples characterized as well as dependant on experimental conditions and techniques [28] used for analysis. Hence, while these proxies may be of value for comparison of samples with similar physical properties (assuming consistency of analytical procedures), their value for comparison of the samples with which this study is concerned is doubtful. Therefore, no attempt has been made to apply or interpret these proxies in the present study.

## Conclusion

It is a widely accepted belief that vitrification occurs gradually over time as part of the overall coalification process and that it is largely temperature-driven. Previous studies discussing this process have generally compared samples collected from different sites and covering a range of maturities [e.g. [29]] and in that sense are actually investigations of the coalification pathway of vitrinite. The focus of the present study is investigation of the structural changes associated with the earliest stages of the formation of vitrinite, i.e. those occurring during the initial conversion of woody plant tissues to vitrinite, and prior to subsequent maturation of the resulting vitrinite.

Samples characterized are all gymnosperm-derived, were collected from the same site, share the same very mild thermal history and were recovered in close proximity to each other. In all cases, cellulosic and hemi-cellulosic materials have been largely removed, suggesting similar degrees of biological diagenesis and further indicating that residual product, (vitrified or unvitrified) is derived largely or exclusively from lignin. Therefore, observed differences between vitrified and unvitrified samples are not the result of differences in the age, thermal maturity or initial composition of the samples. The key difference between the samples is the degree of physical compression experienced. The vitrified samples were all recovered from coaly layers that have undergone significant compaction and compression due to the weight of the overlying sediments. Unvitrified materials were recovered from unconsolidated sands adjacent to the coaly layers. Sands are much less compressible than peats and hence, materials deposited in these layers were protected from compaction. Therefore, it is the view of the present authors that physical compression is a key driver of the geochemical changes that are associated with the initial vitrification of buried lignin-rich plant tissues.

Comparison of thermochemolysis results from vitrified and unvitrified samples suggests that vitrification is associated with significant changes in the lignin structure. The data suggest that these changes occur primarily by reactions involving and altering the nature of the C3 side-chain unit, reducing the number of β-O-4 linked lignin units. This is consistent with previously reported observations [2,11,29], except that in this study no evidence of the trans-alkylation of C5 of the lignin aromatic unit as previously proposed [11,29] is observed. This is not to imply that such reactions may not subsequently be important during further maturation of the vitrinite. However, given (i) the complete absence of C5-alkyl products in the data reported herein and also (ii) that all of the observed products are now known to be derivable from structures that do not involve C-5 alkylation [15], we conclude that the initial reactions that result in conversion of recognizably woody tissues to vitrinite, occur wholly within the lignin side chain.

## Supplementary Material

Additional File 1 Supporting interactive supplemental data for Figure 4, including machine readable MS data are given in Additional File 1.zip. To access these data, download this file and unzip the compressed archive, ensuring that the embedded directory structure is preserved. Once uncompressed, simply open IDS Figure 4.htm. JavaScript must be enabled in your web browser in order to fully access these files. These files will also be available on line via the Geochemical Transactions web site in the near future. Click here for file

Additional File 2 Supporting interactive supplemental data for Figure 6, including machine readable MS data are given in Additional File 2.zip. To access these data, download this file and unzip the compressed archive, ensuring that the embedded directory structure is preserved. Once uncompressed, simply open IDS Figure 6.htm. JavaScript must be enabled in your web browser in order to fully access these files. These files will also be available on line via the Geochemical Transactions web site in the near future. Click here for file
