# Supplementary figures and images for: Comparison of vitrified and unvitrified Eocene woody tissues by TMAH thermochemolysis – implications for the early stages of the formation of vitrinite (part 1 of 4)
Source: Geochem Trans. 2006 Oct 10;7:9. doi: 10.1186/1467-4866-7-9 (PMC1622741; doi:10.1186/1467-4866-7-9)

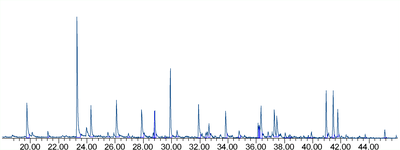

Supplement: Additional File 1 — Supporting interactive supplemental data for Figure 4, including machine readable MS data are given in Additional File 1.zip. To access these data, download this file and unzip the compressed archive, ensuring that the embedded directory structure is preserved. Once uncompressed, simply open IDS Figure 4.htm. JavaScript must be enabled in your web browser in order to fully access these files. These files will also be available on line via the Geochemical Transactions web site in the near future. [file 1467-4866-7-9-S1.zip › Additional File 1/Data1/Chromatograms1/DS1Peak0.png]

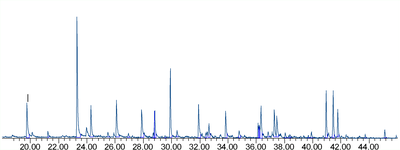

Supplement: Additional File 1 — Supporting interactive supplemental data for Figure 4, including machine readable MS data are given in Additional File 1.zip. To access these data, download this file and unzip the compressed archive, ensuring that the embedded directory structure is preserved. Once uncompressed, simply open IDS Figure 4.htm. JavaScript must be enabled in your web browser in order to fully access these files. These files will also be available on line via the Geochemical Transactions web site in the near future. [file 1467-4866-7-9-S1.zip › Additional File 1/Data1/Chromatograms1/DS1Peak1.png]

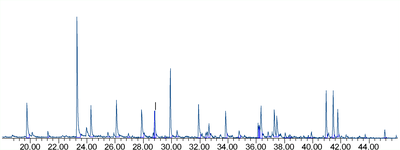

Supplement: Additional File 1 — Supporting interactive supplemental data for Figure 4, including machine readable MS data are given in Additional File 1.zip. To access these data, download this file and unzip the compressed archive, ensuring that the embedded directory structure is preserved. Once uncompressed, simply open IDS Figure 4.htm. JavaScript must be enabled in your web browser in order to fully access these files. These files will also be available on line via the Geochemical Transactions web site in the near future. [file 1467-4866-7-9-S1.zip › Additional File 1/Data1/Chromatograms1/DS1Peak10.png]

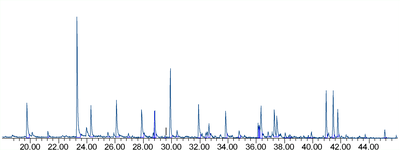

Supplement: Additional File 1 — Supporting interactive supplemental data for Figure 4, including machine readable MS data are given in Additional File 1.zip. To access these data, download this file and unzip the compressed archive, ensuring that the embedded directory structure is preserved. Once uncompressed, simply open IDS Figure 4.htm. JavaScript must be enabled in your web browser in order to fully access these files. These files will also be available on line via the Geochemical Transactions web site in the near future. [file 1467-4866-7-9-S1.zip › Additional File 1/Data1/Chromatograms1/DS1Peak12.png]

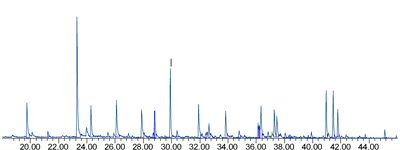

Supplement: Additional File 1 — Supporting interactive supplemental data for Figure 4, including machine readable MS data are given in Additional File 1.zip. To access these data, download this file and unzip the compressed archive, ensuring that the embedded directory structure is preserved. Once uncompressed, simply open IDS Figure 4.htm. JavaScript must be enabled in your web browser in order to fully access these files. These files will also be available on line via the Geochemical Transactions web site in the near future. [file 1467-4866-7-9-S1.zip › Additional File 1/Data1/Chromatograms1/DS1Peak13.png]

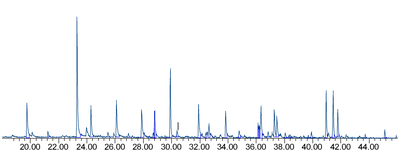

Supplement: Additional File 1 — Supporting interactive supplemental data for Figure 4, including machine readable MS data are given in Additional File 1.zip. To access these data, download this file and unzip the compressed archive, ensuring that the embedded directory structure is preserved. Once uncompressed, simply open IDS Figure 4.htm. JavaScript must be enabled in your web browser in order to fully access these files. These files will also be available on line via the Geochemical Transactions web site in the near future. [file 1467-4866-7-9-S1.zip › Additional File 1/Data1/Chromatograms1/DS1Peak14.png]

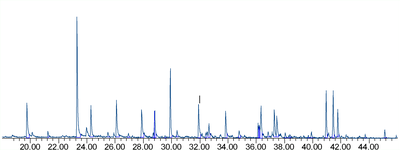

Supplement: Additional File 1 — Supporting interactive supplemental data for Figure 4, including machine readable MS data are given in Additional File 1.zip. To access these data, download this file and unzip the compressed archive, ensuring that the embedded directory structure is preserved. Once uncompressed, simply open IDS Figure 4.htm. JavaScript must be enabled in your web browser in order to fully access these files. These files will also be available on line via the Geochemical Transactions web site in the near future. [file 1467-4866-7-9-S1.zip › Additional File 1/Data1/Chromatograms1/DS1Peak15.png]

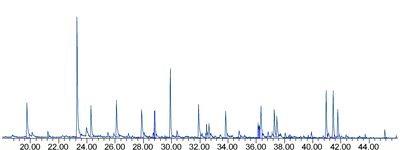

Supplement: Additional File 1 — Supporting interactive supplemental data for Figure 4, including machine readable MS data are given in Additional File 1.zip. To access these data, download this file and unzip the compressed archive, ensuring that the embedded directory structure is preserved. Once uncompressed, simply open IDS Figure 4.htm. JavaScript must be enabled in your web browser in order to fully access these files. These files will also be available on line via the Geochemical Transactions web site in the near future. [file 1467-4866-7-9-S1.zip › Additional File 1/Data1/Chromatograms1/DS1Peak16.png]

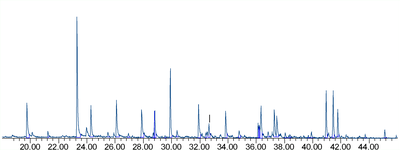

Supplement: Additional File 1 — Supporting interactive supplemental data for Figure 4, including machine readable MS data are given in Additional File 1.zip. To access these data, download this file and unzip the compressed archive, ensuring that the embedded directory structure is preserved. Once uncompressed, simply open IDS Figure 4.htm. JavaScript must be enabled in your web browser in order to fully access these files. These files will also be available on line via the Geochemical Transactions web site in the near future. [file 1467-4866-7-9-S1.zip › Additional File 1/Data1/Chromatograms1/DS1Peak17.png]

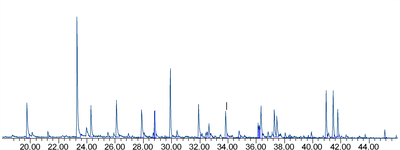

Supplement: Additional File 1 — Supporting interactive supplemental data for Figure 4, including machine readable MS data are given in Additional File 1.zip. To access these data, download this file and unzip the compressed archive, ensuring that the embedded directory structure is preserved. Once uncompressed, simply open IDS Figure 4.htm. JavaScript must be enabled in your web browser in order to fully access these files. These files will also be available on line via the Geochemical Transactions web site in the near future. [file 1467-4866-7-9-S1.zip › Additional File 1/Data1/Chromatograms1/DS1Peak18.png]

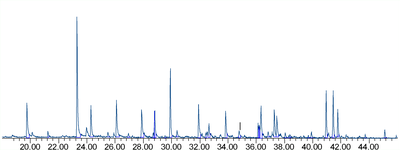

Supplement: Additional File 1 — Supporting interactive supplemental data for Figure 4, including machine readable MS data are given in Additional File 1.zip. To access these data, download this file and unzip the compressed archive, ensuring that the embedded directory structure is preserved. Once uncompressed, simply open IDS Figure 4.htm. JavaScript must be enabled in your web browser in order to fully access these files. These files will also be available on line via the Geochemical Transactions web site in the near future. [file 1467-4866-7-9-S1.zip › Additional File 1/Data1/Chromatograms1/DS1Peak19.png]

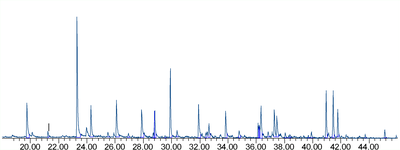

Supplement: Additional File 1 — Supporting interactive supplemental data for Figure 4, including machine readable MS data are given in Additional File 1.zip. To access these data, download this file and unzip the compressed archive, ensuring that the embedded directory structure is preserved. Once uncompressed, simply open IDS Figure 4.htm. JavaScript must be enabled in your web browser in order to fully access these files. These files will also be available on line via the Geochemical Transactions web site in the near future. [file 1467-4866-7-9-S1.zip › Additional File 1/Data1/Chromatograms1/DS1Peak2.png]

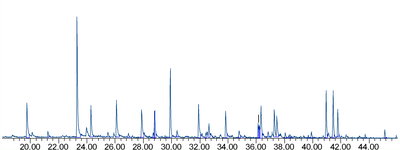

Supplement: Additional File 1 — Supporting interactive supplemental data for Figure 4, including machine readable MS data are given in Additional File 1.zip. To access these data, download this file and unzip the compressed archive, ensuring that the embedded directory structure is preserved. Once uncompressed, simply open IDS Figure 4.htm. JavaScript must be enabled in your web browser in order to fully access these files. These files will also be available on line via the Geochemical Transactions web site in the near future. [file 1467-4866-7-9-S1.zip › Additional File 1/Data1/Chromatograms1/DS1Peak20.png]

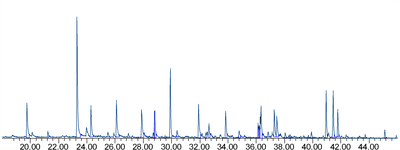

Supplement: Additional File 1 — Supporting interactive supplemental data for Figure 4, including machine readable MS data are given in Additional File 1.zip. To access these data, download this file and unzip the compressed archive, ensuring that the embedded directory structure is preserved. Once uncompressed, simply open IDS Figure 4.htm. JavaScript must be enabled in your web browser in order to fully access these files. These files will also be available on line via the Geochemical Transactions web site in the near future. [file 1467-4866-7-9-S1.zip › Additional File 1/Data1/Chromatograms1/DS1Peak21.png]

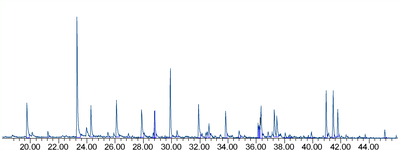

Supplement: Additional File 1 — Supporting interactive supplemental data for Figure 4, including machine readable MS data are given in Additional File 1.zip. To access these data, download this file and unzip the compressed archive, ensuring that the embedded directory structure is preserved. Once uncompressed, simply open IDS Figure 4.htm. JavaScript must be enabled in your web browser in order to fully access these files. These files will also be available on line via the Geochemical Transactions web site in the near future. [file 1467-4866-7-9-S1.zip › Additional File 1/Data1/Chromatograms1/DS1Peak22.png]

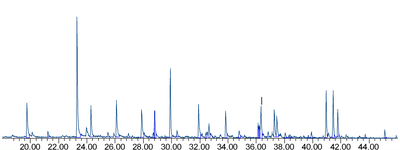

Supplement: Additional File 1 — Supporting interactive supplemental data for Figure 4, including machine readable MS data are given in Additional File 1.zip. To access these data, download this file and unzip the compressed archive, ensuring that the embedded directory structure is preserved. Once uncompressed, simply open IDS Figure 4.htm. JavaScript must be enabled in your web browser in order to fully access these files. These files will also be available on line via the Geochemical Transactions web site in the near future. [file 1467-4866-7-9-S1.zip › Additional File 1/Data1/Chromatograms1/DS1Peak23.png]

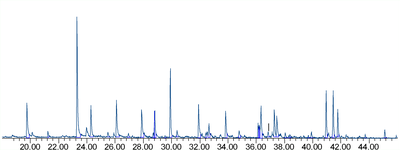

Supplement: Additional File 1 — Supporting interactive supplemental data for Figure 4, including machine readable MS data are given in Additional File 1.zip. To access these data, download this file and unzip the compressed archive, ensuring that the embedded directory structure is preserved. Once uncompressed, simply open IDS Figure 4.htm. JavaScript must be enabled in your web browser in order to fully access these files. These files will also be available on line via the Geochemical Transactions web site in the near future. [file 1467-4866-7-9-S1.zip › Additional File 1/Data1/Chromatograms1/DS1Peak24.png]

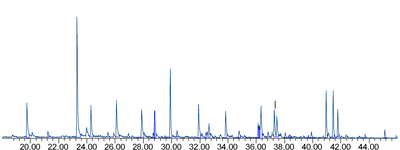

Supplement: Additional File 1 — Supporting interactive supplemental data for Figure 4, including machine readable MS data are given in Additional File 1.zip. To access these data, download this file and unzip the compressed archive, ensuring that the embedded directory structure is preserved. Once uncompressed, simply open IDS Figure 4.htm. JavaScript must be enabled in your web browser in order to fully access these files. These files will also be available on line via the Geochemical Transactions web site in the near future. [file 1467-4866-7-9-S1.zip › Additional File 1/Data1/Chromatograms1/DS1Peak25.png]

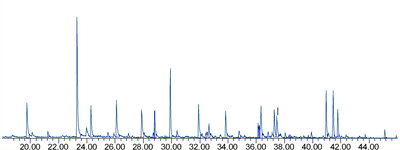

Supplement: Additional File 1 — Supporting interactive supplemental data for Figure 4, including machine readable MS data are given in Additional File 1.zip. To access these data, download this file and unzip the compressed archive, ensuring that the embedded directory structure is preserved. Once uncompressed, simply open IDS Figure 4.htm. JavaScript must be enabled in your web browser in order to fully access these files. These files will also be available on line via the Geochemical Transactions web site in the near future. [file 1467-4866-7-9-S1.zip › Additional File 1/Data1/Chromatograms1/DS1Peak26.png]

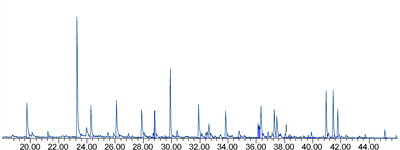

Supplement: Additional File 1 — Supporting interactive supplemental data for Figure 4, including machine readable MS data are given in Additional File 1.zip. To access these data, download this file and unzip the compressed archive, ensuring that the embedded directory structure is preserved. Once uncompressed, simply open IDS Figure 4.htm. JavaScript must be enabled in your web browser in order to fully access these files. These files will also be available on line via the Geochemical Transactions web site in the near future. [file 1467-4866-7-9-S1.zip › Additional File 1/Data1/Chromatograms1/DS1Peak27.png]

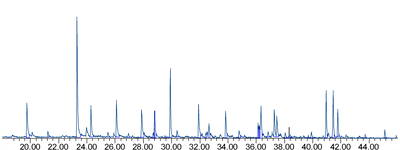

Supplement: Additional File 1 — Supporting interactive supplemental data for Figure 4, including machine readable MS data are given in Additional File 1.zip. To access these data, download this file and unzip the compressed archive, ensuring that the embedded directory structure is preserved. Once uncompressed, simply open IDS Figure 4.htm. JavaScript must be enabled in your web browser in order to fully access these files. These files will also be available on line via the Geochemical Transactions web site in the near future. [file 1467-4866-7-9-S1.zip › Additional File 1/Data1/Chromatograms1/DS1Peak28.png]

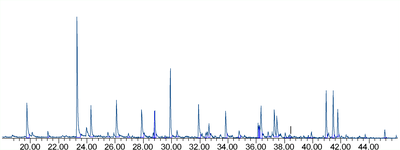

Supplement: Additional File 1 — Supporting interactive supplemental data for Figure 4, including machine readable MS data are given in Additional File 1.zip. To access these data, download this file and unzip the compressed archive, ensuring that the embedded directory structure is preserved. Once uncompressed, simply open IDS Figure 4.htm. JavaScript must be enabled in your web browser in order to fully access these files. These files will also be available on line via the Geochemical Transactions web site in the near future. [file 1467-4866-7-9-S1.zip › Additional File 1/Data1/Chromatograms1/DS1Peak29.png]

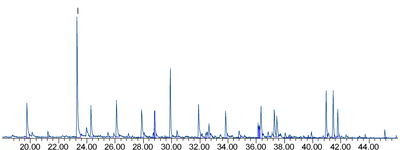

Supplement: Additional File 1 — Supporting interactive supplemental data for Figure 4, including machine readable MS data are given in Additional File 1.zip. To access these data, download this file and unzip the compressed archive, ensuring that the embedded directory structure is preserved. Once uncompressed, simply open IDS Figure 4.htm. JavaScript must be enabled in your web browser in order to fully access these files. These files will also be available on line via the Geochemical Transactions web site in the near future. [file 1467-4866-7-9-S1.zip › Additional File 1/Data1/Chromatograms1/DS1Peak3.png]

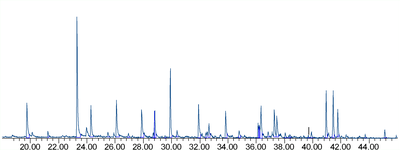

Supplement: Additional File 1 — Supporting interactive supplemental data for Figure 4, including machine readable MS data are given in Additional File 1.zip. To access these data, download this file and unzip the compressed archive, ensuring that the embedded directory structure is preserved. Once uncompressed, simply open IDS Figure 4.htm. JavaScript must be enabled in your web browser in order to fully access these files. These files will also be available on line via the Geochemical Transactions web site in the near future. [file 1467-4866-7-9-S1.zip › Additional File 1/Data1/Chromatograms1/DS1Peak30.png]

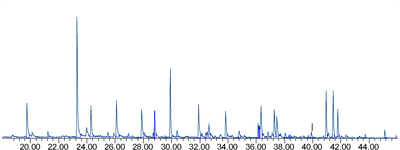

Supplement: Additional File 1 — Supporting interactive supplemental data for Figure 4, including machine readable MS data are given in Additional File 1.zip. To access these data, download this file and unzip the compressed archive, ensuring that the embedded directory structure is preserved. Once uncompressed, simply open IDS Figure 4.htm. JavaScript must be enabled in your web browser in order to fully access these files. These files will also be available on line via the Geochemical Transactions web site in the near future. [file 1467-4866-7-9-S1.zip › Additional File 1/Data1/Chromatograms1/DS1Peak31.png]

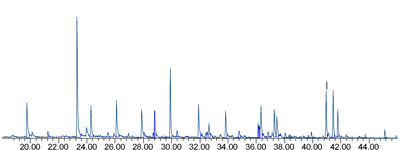

Supplement: Additional File 1 — Supporting interactive supplemental data for Figure 4, including machine readable MS data are given in Additional File 1.zip. To access these data, download this file and unzip the compressed archive, ensuring that the embedded directory structure is preserved. Once uncompressed, simply open IDS Figure 4.htm. JavaScript must be enabled in your web browser in order to fully access these files. These files will also be available on line via the Geochemical Transactions web site in the near future. [file 1467-4866-7-9-S1.zip › Additional File 1/Data1/Chromatograms1/DS1Peak32.png]

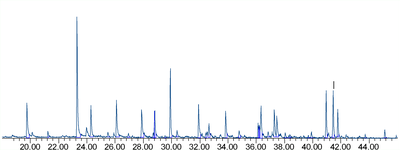

Supplement: Additional File 1 — Supporting interactive supplemental data for Figure 4, including machine readable MS data are given in Additional File 1.zip. To access these data, download this file and unzip the compressed archive, ensuring that the embedded directory structure is preserved. Once uncompressed, simply open IDS Figure 4.htm. JavaScript must be enabled in your web browser in order to fully access these files. These files will also be available on line via the Geochemical Transactions web site in the near future. [file 1467-4866-7-9-S1.zip › Additional File 1/Data1/Chromatograms1/DS1Peak33.png]

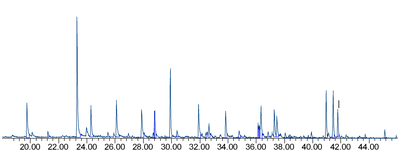

Supplement: Additional File 1 — Supporting interactive supplemental data for Figure 4, including machine readable MS data are given in Additional File 1.zip. To access these data, download this file and unzip the compressed archive, ensuring that the embedded directory structure is preserved. Once uncompressed, simply open IDS Figure 4.htm. JavaScript must be enabled in your web browser in order to fully access these files. These files will also be available on line via the Geochemical Transactions web site in the near future. [file 1467-4866-7-9-S1.zip › Additional File 1/Data1/Chromatograms1/DS1Peak34.png]

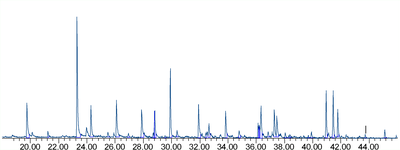

Supplement: Additional File 1 — Supporting interactive supplemental data for Figure 4, including machine readable MS data are given in Additional File 1.zip. To access these data, download this file and unzip the compressed archive, ensuring that the embedded directory structure is preserved. Once uncompressed, simply open IDS Figure 4.htm. JavaScript must be enabled in your web browser in order to fully access these files. These files will also be available on line via the Geochemical Transactions web site in the near future. [file 1467-4866-7-9-S1.zip › Additional File 1/Data1/Chromatograms1/DS1Peak35.png]

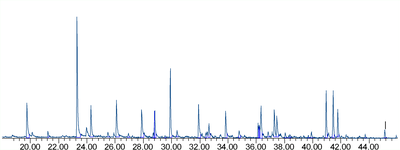

Supplement: Additional File 1 — Supporting interactive supplemental data for Figure 4, including machine readable MS data are given in Additional File 1.zip. To access these data, download this file and unzip the compressed archive, ensuring that the embedded directory structure is preserved. Once uncompressed, simply open IDS Figure 4.htm. JavaScript must be enabled in your web browser in order to fully access these files. These files will also be available on line via the Geochemical Transactions web site in the near future. [file 1467-4866-7-9-S1.zip › Additional File 1/Data1/Chromatograms1/DS1Peak36.png]

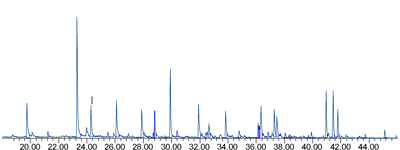

Supplement: Additional File 1 — Supporting interactive supplemental data for Figure 4, including machine readable MS data are given in Additional File 1.zip. To access these data, download this file and unzip the compressed archive, ensuring that the embedded directory structure is preserved. Once uncompressed, simply open IDS Figure 4.htm. JavaScript must be enabled in your web browser in order to fully access these files. These files will also be available on line via the Geochemical Transactions web site in the near future. [file 1467-4866-7-9-S1.zip › Additional File 1/Data1/Chromatograms1/DS1Peak4.png]

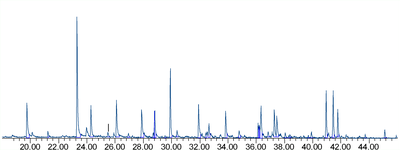

Supplement: Additional File 1 — Supporting interactive supplemental data for Figure 4, including machine readable MS data are given in Additional File 1.zip. To access these data, download this file and unzip the compressed archive, ensuring that the embedded directory structure is preserved. Once uncompressed, simply open IDS Figure 4.htm. JavaScript must be enabled in your web browser in order to fully access these files. These files will also be available on line via the Geochemical Transactions web site in the near future. [file 1467-4866-7-9-S1.zip › Additional File 1/Data1/Chromatograms1/DS1Peak5.png]

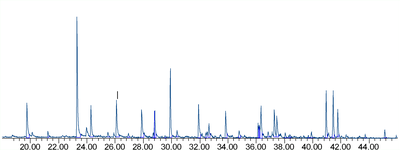

Supplement: Additional File 1 — Supporting interactive supplemental data for Figure 4, including machine readable MS data are given in Additional File 1.zip. To access these data, download this file and unzip the compressed archive, ensuring that the embedded directory structure is preserved. Once uncompressed, simply open IDS Figure 4.htm. JavaScript must be enabled in your web browser in order to fully access these files. These files will also be available on line via the Geochemical Transactions web site in the near future. [file 1467-4866-7-9-S1.zip › Additional File 1/Data1/Chromatograms1/DS1Peak6.png]

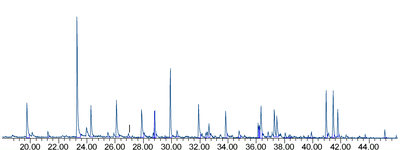

Supplement: Additional File 1 — Supporting interactive supplemental data for Figure 4, including machine readable MS data are given in Additional File 1.zip. To access these data, download this file and unzip the compressed archive, ensuring that the embedded directory structure is preserved. Once uncompressed, simply open IDS Figure 4.htm. JavaScript must be enabled in your web browser in order to fully access these files. These files will also be available on line via the Geochemical Transactions web site in the near future. [file 1467-4866-7-9-S1.zip › Additional File 1/Data1/Chromatograms1/DS1Peak7.png]

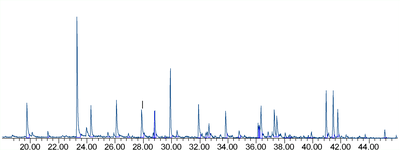

Supplement: Additional File 1 — Supporting interactive supplemental data for Figure 4, including machine readable MS data are given in Additional File 1.zip. To access these data, download this file and unzip the compressed archive, ensuring that the embedded directory structure is preserved. Once uncompressed, simply open IDS Figure 4.htm. JavaScript must be enabled in your web browser in order to fully access these files. These files will also be available on line via the Geochemical Transactions web site in the near future. [file 1467-4866-7-9-S1.zip › Additional File 1/Data1/Chromatograms1/DS1Peak8.png]

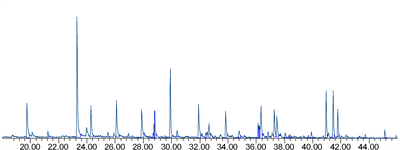

Supplement: Additional File 1 — Supporting interactive supplemental data for Figure 4, including machine readable MS data are given in Additional File 1.zip. To access these data, download this file and unzip the compressed archive, ensuring that the embedded directory structure is preserved. Once uncompressed, simply open IDS Figure 4.htm. JavaScript must be enabled in your web browser in order to fully access these files. These files will also be available on line via the Geochemical Transactions web site in the near future. [file 1467-4866-7-9-S1.zip › Additional File 1/Data1/Chromatograms1/DS1Peak9.png]

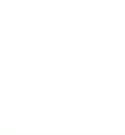

Supplement: Additional File 1 — Supporting interactive supplemental data for Figure 4, including machine readable MS data are given in Additional File 1.zip. To access these data, download this file and unzip the compressed archive, ensuring that the embedded directory structure is preserved. Once uncompressed, simply open IDS Figure 4.htm. JavaScript must be enabled in your web browser in order to fully access these files. These files will also be available on line via the Geochemical Transactions web site in the near future. [file 1467-4866-7-9-S1.zip › Additional File 1/Data1/Chromatograms1/Peaks1/Pk1-0.png]

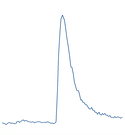

Supplement: Additional File 1 — Supporting interactive supplemental data for Figure 4, including machine readable MS data are given in Additional File 1.zip. To access these data, download this file and unzip the compressed archive, ensuring that the embedded directory structure is preserved. Once uncompressed, simply open IDS Figure 4.htm. JavaScript must be enabled in your web browser in order to fully access these files. These files will also be available on line via the Geochemical Transactions web site in the near future. [file 1467-4866-7-9-S1.zip › Additional File 1/Data1/Chromatograms1/Peaks1/Pk1-1.png]

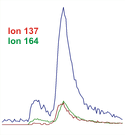

Supplement: Additional File 1 — Supporting interactive supplemental data for Figure 4, including machine readable MS data are given in Additional File 1.zip. To access these data, download this file and unzip the compressed archive, ensuring that the embedded directory structure is preserved. Once uncompressed, simply open IDS Figure 4.htm. JavaScript must be enabled in your web browser in order to fully access these files. These files will also be available on line via the Geochemical Transactions web site in the near future. [file 1467-4866-7-9-S1.zip › Additional File 1/Data1/Chromatograms1/Peaks1/Pk1-10.png]

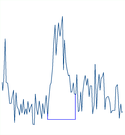

Supplement: Additional File 1 — Supporting interactive supplemental data for Figure 4, including machine readable MS data are given in Additional File 1.zip. To access these data, download this file and unzip the compressed archive, ensuring that the embedded directory structure is preserved. Once uncompressed, simply open IDS Figure 4.htm. JavaScript must be enabled in your web browser in order to fully access these files. These files will also be available on line via the Geochemical Transactions web site in the near future. [file 1467-4866-7-9-S1.zip › Additional File 1/Data1/Chromatograms1/Peaks1/Pk1-12.png]

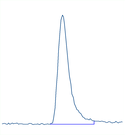

Supplement: Additional File 1 — Supporting interactive supplemental data for Figure 4, including machine readable MS data are given in Additional File 1.zip. To access these data, download this file and unzip the compressed archive, ensuring that the embedded directory structure is preserved. Once uncompressed, simply open IDS Figure 4.htm. JavaScript must be enabled in your web browser in order to fully access these files. These files will also be available on line via the Geochemical Transactions web site in the near future. [file 1467-4866-7-9-S1.zip › Additional File 1/Data1/Chromatograms1/Peaks1/Pk1-13.png]

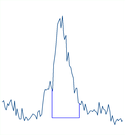

Supplement: Additional File 1 — Supporting interactive supplemental data for Figure 4, including machine readable MS data are given in Additional File 1.zip. To access these data, download this file and unzip the compressed archive, ensuring that the embedded directory structure is preserved. Once uncompressed, simply open IDS Figure 4.htm. JavaScript must be enabled in your web browser in order to fully access these files. These files will also be available on line via the Geochemical Transactions web site in the near future. [file 1467-4866-7-9-S1.zip › Additional File 1/Data1/Chromatograms1/Peaks1/Pk1-14.png]

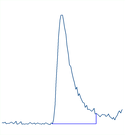

Supplement: Additional File 1 — Supporting interactive supplemental data for Figure 4, including machine readable MS data are given in Additional File 1.zip. To access these data, download this file and unzip the compressed archive, ensuring that the embedded directory structure is preserved. Once uncompressed, simply open IDS Figure 4.htm. JavaScript must be enabled in your web browser in order to fully access these files. These files will also be available on line via the Geochemical Transactions web site in the near future. [file 1467-4866-7-9-S1.zip › Additional File 1/Data1/Chromatograms1/Peaks1/Pk1-15.png]

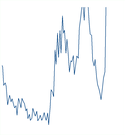

Supplement: Additional File 1 — Supporting interactive supplemental data for Figure 4, including machine readable MS data are given in Additional File 1.zip. To access these data, download this file and unzip the compressed archive, ensuring that the embedded directory structure is preserved. Once uncompressed, simply open IDS Figure 4.htm. JavaScript must be enabled in your web browser in order to fully access these files. These files will also be available on line via the Geochemical Transactions web site in the near future. [file 1467-4866-7-9-S1.zip › Additional File 1/Data1/Chromatograms1/Peaks1/Pk1-16.png]

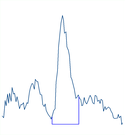

Supplement: Additional File 1 — Supporting interactive supplemental data for Figure 4, including machine readable MS data are given in Additional File 1.zip. To access these data, download this file and unzip the compressed archive, ensuring that the embedded directory structure is preserved. Once uncompressed, simply open IDS Figure 4.htm. JavaScript must be enabled in your web browser in order to fully access these files. These files will also be available on line via the Geochemical Transactions web site in the near future. [file 1467-4866-7-9-S1.zip › Additional File 1/Data1/Chromatograms1/Peaks1/Pk1-17.png]

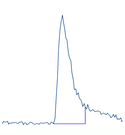

Supplement: Additional File 1 — Supporting interactive supplemental data for Figure 4, including machine readable MS data are given in Additional File 1.zip. To access these data, download this file and unzip the compressed archive, ensuring that the embedded directory structure is preserved. Once uncompressed, simply open IDS Figure 4.htm. JavaScript must be enabled in your web browser in order to fully access these files. These files will also be available on line via the Geochemical Transactions web site in the near future. [file 1467-4866-7-9-S1.zip › Additional File 1/Data1/Chromatograms1/Peaks1/Pk1-18.png]

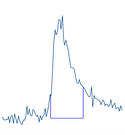

Supplement: Additional File 1 — Supporting interactive supplemental data for Figure 4, including machine readable MS data are given in Additional File 1.zip. To access these data, download this file and unzip the compressed archive, ensuring that the embedded directory structure is preserved. Once uncompressed, simply open IDS Figure 4.htm. JavaScript must be enabled in your web browser in order to fully access these files. These files will also be available on line via the Geochemical Transactions web site in the near future. [file 1467-4866-7-9-S1.zip › Additional File 1/Data1/Chromatograms1/Peaks1/Pk1-19.png]

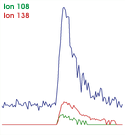

Supplement: Additional File 1 — Supporting interactive supplemental data for Figure 4, including machine readable MS data are given in Additional File 1.zip. To access these data, download this file and unzip the compressed archive, ensuring that the embedded directory structure is preserved. Once uncompressed, simply open IDS Figure 4.htm. JavaScript must be enabled in your web browser in order to fully access these files. These files will also be available on line via the Geochemical Transactions web site in the near future. [file 1467-4866-7-9-S1.zip › Additional File 1/Data1/Chromatograms1/Peaks1/Pk1-2.png]

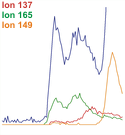

Supplement: Additional File 1 — Supporting interactive supplemental data for Figure 4, including machine readable MS data are given in Additional File 1.zip. To access these data, download this file and unzip the compressed archive, ensuring that the embedded directory structure is preserved. Once uncompressed, simply open IDS Figure 4.htm. JavaScript must be enabled in your web browser in order to fully access these files. These files will also be available on line via the Geochemical Transactions web site in the near future. [file 1467-4866-7-9-S1.zip › Additional File 1/Data1/Chromatograms1/Peaks1/Pk1-20.png]

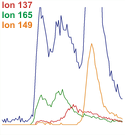

Supplement: Additional File 1 — Supporting interactive supplemental data for Figure 4, including machine readable MS data are given in Additional File 1.zip. To access these data, download this file and unzip the compressed archive, ensuring that the embedded directory structure is preserved. Once uncompressed, simply open IDS Figure 4.htm. JavaScript must be enabled in your web browser in order to fully access these files. These files will also be available on line via the Geochemical Transactions web site in the near future. [file 1467-4866-7-9-S1.zip › Additional File 1/Data1/Chromatograms1/Peaks1/Pk1-21.png]

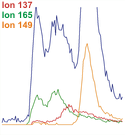

Supplement: Additional File 1 — Supporting interactive supplemental data for Figure 4, including machine readable MS data are given in Additional File 1.zip. To access these data, download this file and unzip the compressed archive, ensuring that the embedded directory structure is preserved. Once uncompressed, simply open IDS Figure 4.htm. JavaScript must be enabled in your web browser in order to fully access these files. These files will also be available on line via the Geochemical Transactions web site in the near future. [file 1467-4866-7-9-S1.zip › Additional File 1/Data1/Chromatograms1/Peaks1/Pk1-22.png]

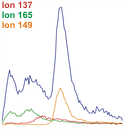

Supplement: Additional File 1 — Supporting interactive supplemental data for Figure 4, including machine readable MS data are given in Additional File 1.zip. To access these data, download this file and unzip the compressed archive, ensuring that the embedded directory structure is preserved. Once uncompressed, simply open IDS Figure 4.htm. JavaScript must be enabled in your web browser in order to fully access these files. These files will also be available on line via the Geochemical Transactions web site in the near future. [file 1467-4866-7-9-S1.zip › Additional File 1/Data1/Chromatograms1/Peaks1/Pk1-23.png]

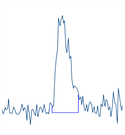

Supplement: Additional File 1 — Supporting interactive supplemental data for Figure 4, including machine readable MS data are given in Additional File 1.zip. To access these data, download this file and unzip the compressed archive, ensuring that the embedded directory structure is preserved. Once uncompressed, simply open IDS Figure 4.htm. JavaScript must be enabled in your web browser in order to fully access these files. These files will also be available on line via the Geochemical Transactions web site in the near future. [file 1467-4866-7-9-S1.zip › Additional File 1/Data1/Chromatograms1/Peaks1/Pk1-24.png]

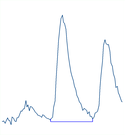

Supplement: Additional File 1 — Supporting interactive supplemental data for Figure 4, including machine readable MS data are given in Additional File 1.zip. To access these data, download this file and unzip the compressed archive, ensuring that the embedded directory structure is preserved. Once uncompressed, simply open IDS Figure 4.htm. JavaScript must be enabled in your web browser in order to fully access these files. These files will also be available on line via the Geochemical Transactions web site in the near future. [file 1467-4866-7-9-S1.zip › Additional File 1/Data1/Chromatograms1/Peaks1/Pk1-25.png]

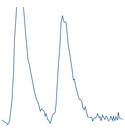

Supplement: Additional File 1 — Supporting interactive supplemental data for Figure 4, including machine readable MS data are given in Additional File 1.zip. To access these data, download this file and unzip the compressed archive, ensuring that the embedded directory structure is preserved. Once uncompressed, simply open IDS Figure 4.htm. JavaScript must be enabled in your web browser in order to fully access these files. These files will also be available on line via the Geochemical Transactions web site in the near future. [file 1467-4866-7-9-S1.zip › Additional File 1/Data1/Chromatograms1/Peaks1/Pk1-26.png]

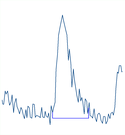

Supplement: Additional File 1 — Supporting interactive supplemental data for Figure 4, including machine readable MS data are given in Additional File 1.zip. To access these data, download this file and unzip the compressed archive, ensuring that the embedded directory structure is preserved. Once uncompressed, simply open IDS Figure 4.htm. JavaScript must be enabled in your web browser in order to fully access these files. These files will also be available on line via the Geochemical Transactions web site in the near future. [file 1467-4866-7-9-S1.zip › Additional File 1/Data1/Chromatograms1/Peaks1/Pk1-27.png]

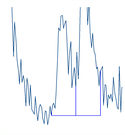

Supplement: Additional File 1 — Supporting interactive supplemental data for Figure 4, including machine readable MS data are given in Additional File 1.zip. To access these data, download this file and unzip the compressed archive, ensuring that the embedded directory structure is preserved. Once uncompressed, simply open IDS Figure 4.htm. JavaScript must be enabled in your web browser in order to fully access these files. These files will also be available on line via the Geochemical Transactions web site in the near future. [file 1467-4866-7-9-S1.zip › Additional File 1/Data1/Chromatograms1/Peaks1/Pk1-28.png]

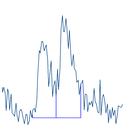

Supplement: Additional File 1 — Supporting interactive supplemental data for Figure 4, including machine readable MS data are given in Additional File 1.zip. To access these data, download this file and unzip the compressed archive, ensuring that the embedded directory structure is preserved. Once uncompressed, simply open IDS Figure 4.htm. JavaScript must be enabled in your web browser in order to fully access these files. These files will also be available on line via the Geochemical Transactions web site in the near future. [file 1467-4866-7-9-S1.zip › Additional File 1/Data1/Chromatograms1/Peaks1/Pk1-29.png]

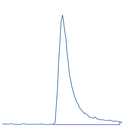

Supplement: Additional File 1 — Supporting interactive supplemental data for Figure 4, including machine readable MS data are given in Additional File 1.zip. To access these data, download this file and unzip the compressed archive, ensuring that the embedded directory structure is preserved. Once uncompressed, simply open IDS Figure 4.htm. JavaScript must be enabled in your web browser in order to fully access these files. These files will also be available on line via the Geochemical Transactions web site in the near future. [file 1467-4866-7-9-S1.zip › Additional File 1/Data1/Chromatograms1/Peaks1/Pk1-3.png]

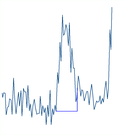

Supplement: Additional File 1 — Supporting interactive supplemental data for Figure 4, including machine readable MS data are given in Additional File 1.zip. To access these data, download this file and unzip the compressed archive, ensuring that the embedded directory structure is preserved. Once uncompressed, simply open IDS Figure 4.htm. JavaScript must be enabled in your web browser in order to fully access these files. These files will also be available on line via the Geochemical Transactions web site in the near future. [file 1467-4866-7-9-S1.zip › Additional File 1/Data1/Chromatograms1/Peaks1/Pk1-30.png]

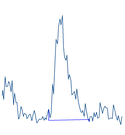

Supplement: Additional File 1 — Supporting interactive supplemental data for Figure 4, including machine readable MS data are given in Additional File 1.zip. To access these data, download this file and unzip the compressed archive, ensuring that the embedded directory structure is preserved. Once uncompressed, simply open IDS Figure 4.htm. JavaScript must be enabled in your web browser in order to fully access these files. These files will also be available on line via the Geochemical Transactions web site in the near future. [file 1467-4866-7-9-S1.zip › Additional File 1/Data1/Chromatograms1/Peaks1/Pk1-31.png]

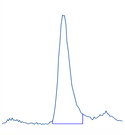

Supplement: Additional File 1 — Supporting interactive supplemental data for Figure 4, including machine readable MS data are given in Additional File 1.zip. To access these data, download this file and unzip the compressed archive, ensuring that the embedded directory structure is preserved. Once uncompressed, simply open IDS Figure 4.htm. JavaScript must be enabled in your web browser in order to fully access these files. These files will also be available on line via the Geochemical Transactions web site in the near future. [file 1467-4866-7-9-S1.zip › Additional File 1/Data1/Chromatograms1/Peaks1/Pk1-32.png]

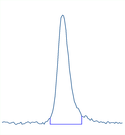

Supplement: Additional File 1 — Supporting interactive supplemental data for Figure 4, including machine readable MS data are given in Additional File 1.zip. To access these data, download this file and unzip the compressed archive, ensuring that the embedded directory structure is preserved. Once uncompressed, simply open IDS Figure 4.htm. JavaScript must be enabled in your web browser in order to fully access these files. These files will also be available on line via the Geochemical Transactions web site in the near future. [file 1467-4866-7-9-S1.zip › Additional File 1/Data1/Chromatograms1/Peaks1/Pk1-33.png]

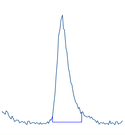

Supplement: Additional File 1 — Supporting interactive supplemental data for Figure 4, including machine readable MS data are given in Additional File 1.zip. To access these data, download this file and unzip the compressed archive, ensuring that the embedded directory structure is preserved. Once uncompressed, simply open IDS Figure 4.htm. JavaScript must be enabled in your web browser in order to fully access these files. These files will also be available on line via the Geochemical Transactions web site in the near future. [file 1467-4866-7-9-S1.zip › Additional File 1/Data1/Chromatograms1/Peaks1/Pk1-34.png]

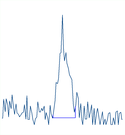

Supplement: Additional File 1 — Supporting interactive supplemental data for Figure 4, including machine readable MS data are given in Additional File 1.zip. To access these data, download this file and unzip the compressed archive, ensuring that the embedded directory structure is preserved. Once uncompressed, simply open IDS Figure 4.htm. JavaScript must be enabled in your web browser in order to fully access these files. These files will also be available on line via the Geochemical Transactions web site in the near future. [file 1467-4866-7-9-S1.zip › Additional File 1/Data1/Chromatograms1/Peaks1/Pk1-35.png]

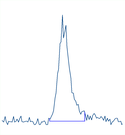

Supplement: Additional File 1 — Supporting interactive supplemental data for Figure 4, including machine readable MS data are given in Additional File 1.zip. To access these data, download this file and unzip the compressed archive, ensuring that the embedded directory structure is preserved. Once uncompressed, simply open IDS Figure 4.htm. JavaScript must be enabled in your web browser in order to fully access these files. These files will also be available on line via the Geochemical Transactions web site in the near future. [file 1467-4866-7-9-S1.zip › Additional File 1/Data1/Chromatograms1/Peaks1/Pk1-36.png]

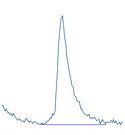

Supplement: Additional File 1 — Supporting interactive supplemental data for Figure 4, including machine readable MS data are given in Additional File 1.zip. To access these data, download this file and unzip the compressed archive, ensuring that the embedded directory structure is preserved. Once uncompressed, simply open IDS Figure 4.htm. JavaScript must be enabled in your web browser in order to fully access these files. These files will also be available on line via the Geochemical Transactions web site in the near future. [file 1467-4866-7-9-S1.zip › Additional File 1/Data1/Chromatograms1/Peaks1/Pk1-4.png]

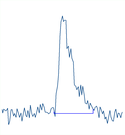

Supplement: Additional File 1 — Supporting interactive supplemental data for Figure 4, including machine readable MS data are given in Additional File 1.zip. To access these data, download this file and unzip the compressed archive, ensuring that the embedded directory structure is preserved. Once uncompressed, simply open IDS Figure 4.htm. JavaScript must be enabled in your web browser in order to fully access these files. These files will also be available on line via the Geochemical Transactions web site in the near future. [file 1467-4866-7-9-S1.zip › Additional File 1/Data1/Chromatograms1/Peaks1/Pk1-5.png]

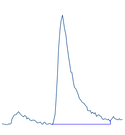

Supplement: Additional File 1 — Supporting interactive supplemental data for Figure 4, including machine readable MS data are given in Additional File 1.zip. To access these data, download this file and unzip the compressed archive, ensuring that the embedded directory structure is preserved. Once uncompressed, simply open IDS Figure 4.htm. JavaScript must be enabled in your web browser in order to fully access these files. These files will also be available on line via the Geochemical Transactions web site in the near future. [file 1467-4866-7-9-S1.zip › Additional File 1/Data1/Chromatograms1/Peaks1/Pk1-6.png]

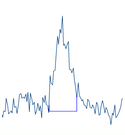

Supplement: Additional File 1 — Supporting interactive supplemental data for Figure 4, including machine readable MS data are given in Additional File 1.zip. To access these data, download this file and unzip the compressed archive, ensuring that the embedded directory structure is preserved. Once uncompressed, simply open IDS Figure 4.htm. JavaScript must be enabled in your web browser in order to fully access these files. These files will also be available on line via the Geochemical Transactions web site in the near future. [file 1467-4866-7-9-S1.zip › Additional File 1/Data1/Chromatograms1/Peaks1/Pk1-7.png]

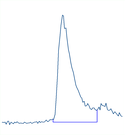

Supplement: Additional File 1 — Supporting interactive supplemental data for Figure 4, including machine readable MS data are given in Additional File 1.zip. To access these data, download this file and unzip the compressed archive, ensuring that the embedded directory structure is preserved. Once uncompressed, simply open IDS Figure 4.htm. JavaScript must be enabled in your web browser in order to fully access these files. These files will also be available on line via the Geochemical Transactions web site in the near future. [file 1467-4866-7-9-S1.zip › Additional File 1/Data1/Chromatograms1/Peaks1/Pk1-8.png]

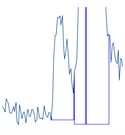

Supplement: Additional File 1 — Supporting interactive supplemental data for Figure 4, including machine readable MS data are given in Additional File 1.zip. To access these data, download this file and unzip the compressed archive, ensuring that the embedded directory structure is preserved. Once uncompressed, simply open IDS Figure 4.htm. JavaScript must be enabled in your web browser in order to fully access these files. These files will also be available on line via the Geochemical Transactions web site in the near future. [file 1467-4866-7-9-S1.zip › Additional File 1/Data1/Chromatograms1/Peaks1/Pk1-9.png]

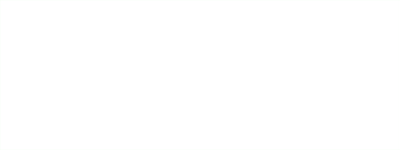

Supplement: Additional File 1 — Supporting interactive supplemental data for Figure 4, including machine readable MS data are given in Additional File 1.zip. To access these data, download this file and unzip the compressed archive, ensuring that the embedded directory structure is preserved. Once uncompressed, simply open IDS Figure 4.htm. JavaScript must be enabled in your web browser in order to fully access these files. These files will also be available on line via the Geochemical Transactions web site in the near future. [file 1467-4866-7-9-S1.zip › Additional File 1/Data1/MSData1/DS1MS0.png]

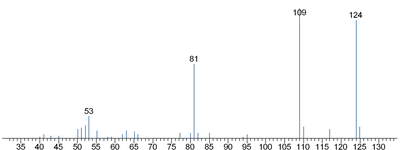

Supplement: Additional File 1 — Supporting interactive supplemental data for Figure 4, including machine readable MS data are given in Additional File 1.zip. To access these data, download this file and unzip the compressed archive, ensuring that the embedded directory structure is preserved. Once uncompressed, simply open IDS Figure 4.htm. JavaScript must be enabled in your web browser in order to fully access these files. These files will also be available on line via the Geochemical Transactions web site in the near future. [file 1467-4866-7-9-S1.zip › Additional File 1/Data1/MSData1/DS1MS1.png]

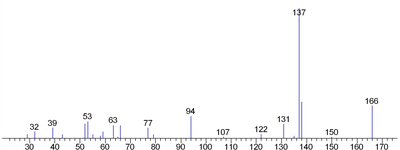

Supplement: Additional File 1 — Supporting interactive supplemental data for Figure 4, including machine readable MS data are given in Additional File 1.zip. To access these data, download this file and unzip the compressed archive, ensuring that the embedded directory structure is preserved. Once uncompressed, simply open IDS Figure 4.htm. JavaScript must be enabled in your web browser in order to fully access these files. These files will also be available on line via the Geochemical Transactions web site in the near future. [file 1467-4866-7-9-S1.zip › Additional File 1/Data1/MSData1/DS1MS10.png]

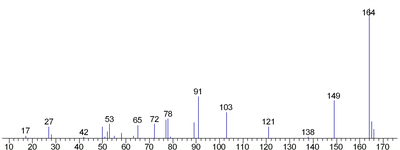

Supplement: Additional File 1 — Supporting interactive supplemental data for Figure 4, including machine readable MS data are given in Additional File 1.zip. To access these data, download this file and unzip the compressed archive, ensuring that the embedded directory structure is preserved. Once uncompressed, simply open IDS Figure 4.htm. JavaScript must be enabled in your web browser in order to fully access these files. These files will also be available on line via the Geochemical Transactions web site in the near future. [file 1467-4866-7-9-S1.zip › Additional File 1/Data1/MSData1/DS1MS11.png]

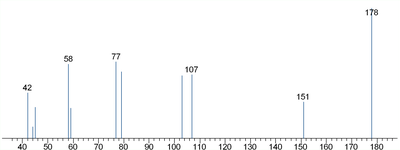

Supplement: Additional File 1 — Supporting interactive supplemental data for Figure 4, including machine readable MS data are given in Additional File 1.zip. To access these data, download this file and unzip the compressed archive, ensuring that the embedded directory structure is preserved. Once uncompressed, simply open IDS Figure 4.htm. JavaScript must be enabled in your web browser in order to fully access these files. These files will also be available on line via the Geochemical Transactions web site in the near future. [file 1467-4866-7-9-S1.zip › Additional File 1/Data1/MSData1/DS1MS12.png]

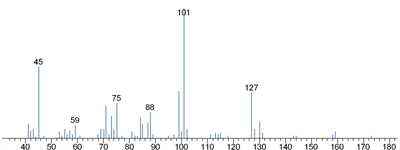

Supplement: Additional File 1 — Supporting interactive supplemental data for Figure 4, including machine readable MS data are given in Additional File 1.zip. To access these data, download this file and unzip the compressed archive, ensuring that the embedded directory structure is preserved. Once uncompressed, simply open IDS Figure 4.htm. JavaScript must be enabled in your web browser in order to fully access these files. These files will also be available on line via the Geochemical Transactions web site in the near future. [file 1467-4866-7-9-S1.zip › Additional File 1/Data1/MSData1/DS1MS13.png]

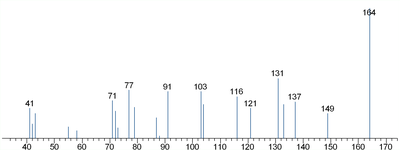

Supplement: Additional File 1 — Supporting interactive supplemental data for Figure 4, including machine readable MS data are given in Additional File 1.zip. To access these data, download this file and unzip the compressed archive, ensuring that the embedded directory structure is preserved. Once uncompressed, simply open IDS Figure 4.htm. JavaScript must be enabled in your web browser in order to fully access these files. These files will also be available on line via the Geochemical Transactions web site in the near future. [file 1467-4866-7-9-S1.zip › Additional File 1/Data1/MSData1/DS1MS14.png]

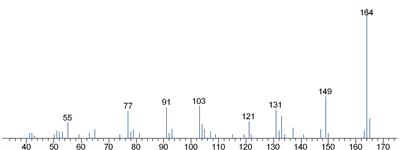

Supplement: Additional File 1 — Supporting interactive supplemental data for Figure 4, including machine readable MS data are given in Additional File 1.zip. To access these data, download this file and unzip the compressed archive, ensuring that the embedded directory structure is preserved. Once uncompressed, simply open IDS Figure 4.htm. JavaScript must be enabled in your web browser in order to fully access these files. These files will also be available on line via the Geochemical Transactions web site in the near future. [file 1467-4866-7-9-S1.zip › Additional File 1/Data1/MSData1/DS1MS15.png]

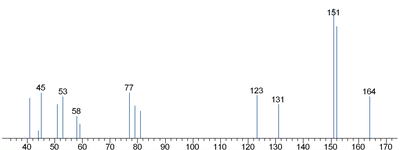

Supplement: Additional File 1 — Supporting interactive supplemental data for Figure 4, including machine readable MS data are given in Additional File 1.zip. To access these data, download this file and unzip the compressed archive, ensuring that the embedded directory structure is preserved. Once uncompressed, simply open IDS Figure 4.htm. JavaScript must be enabled in your web browser in order to fully access these files. These files will also be available on line via the Geochemical Transactions web site in the near future. [file 1467-4866-7-9-S1.zip › Additional File 1/Data1/MSData1/DS1MS16.png]

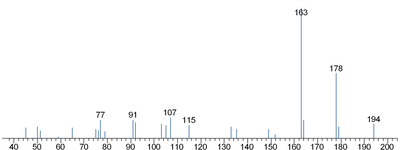

Supplement: Additional File 1 — Supporting interactive supplemental data for Figure 4, including machine readable MS data are given in Additional File 1.zip. To access these data, download this file and unzip the compressed archive, ensuring that the embedded directory structure is preserved. Once uncompressed, simply open IDS Figure 4.htm. JavaScript must be enabled in your web browser in order to fully access these files. These files will also be available on line via the Geochemical Transactions web site in the near future. [file 1467-4866-7-9-S1.zip › Additional File 1/Data1/MSData1/DS1MS17.png]

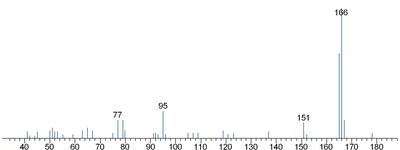

Supplement: Additional File 1 — Supporting interactive supplemental data for Figure 4, including machine readable MS data are given in Additional File 1.zip. To access these data, download this file and unzip the compressed archive, ensuring that the embedded directory structure is preserved. Once uncompressed, simply open IDS Figure 4.htm. JavaScript must be enabled in your web browser in order to fully access these files. These files will also be available on line via the Geochemical Transactions web site in the near future. [file 1467-4866-7-9-S1.zip › Additional File 1/Data1/MSData1/DS1MS18.png]

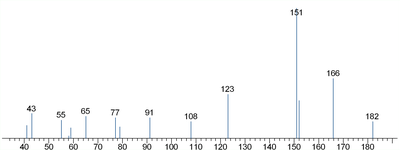

Supplement: Additional File 1 — Supporting interactive supplemental data for Figure 4, including machine readable MS data are given in Additional File 1.zip. To access these data, download this file and unzip the compressed archive, ensuring that the embedded directory structure is preserved. Once uncompressed, simply open IDS Figure 4.htm. JavaScript must be enabled in your web browser in order to fully access these files. These files will also be available on line via the Geochemical Transactions web site in the near future. [file 1467-4866-7-9-S1.zip › Additional File 1/Data1/MSData1/DS1MS19.png]

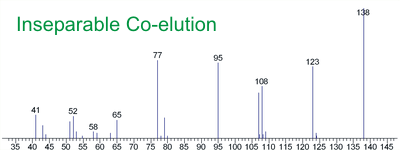

Supplement: Additional File 1 — Supporting interactive supplemental data for Figure 4, including machine readable MS data are given in Additional File 1.zip. To access these data, download this file and unzip the compressed archive, ensuring that the embedded directory structure is preserved. Once uncompressed, simply open IDS Figure 4.htm. JavaScript must be enabled in your web browser in order to fully access these files. These files will also be available on line via the Geochemical Transactions web site in the near future. [file 1467-4866-7-9-S1.zip › Additional File 1/Data1/MSData1/DS1MS2.png]

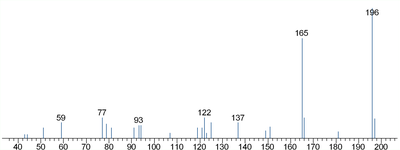

Supplement: Additional File 1 — Supporting interactive supplemental data for Figure 4, including machine readable MS data are given in Additional File 1.zip. To access these data, download this file and unzip the compressed archive, ensuring that the embedded directory structure is preserved. Once uncompressed, simply open IDS Figure 4.htm. JavaScript must be enabled in your web browser in order to fully access these files. These files will also be available on line via the Geochemical Transactions web site in the near future. [file 1467-4866-7-9-S1.zip › Additional File 1/Data1/MSData1/DS1MS20.png]

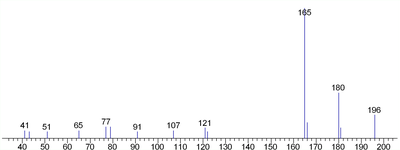

Supplement: Additional File 1 — Supporting interactive supplemental data for Figure 4, including machine readable MS data are given in Additional File 1.zip. To access these data, download this file and unzip the compressed archive, ensuring that the embedded directory structure is preserved. Once uncompressed, simply open IDS Figure 4.htm. JavaScript must be enabled in your web browser in order to fully access these files. These files will also be available on line via the Geochemical Transactions web site in the near future. [file 1467-4866-7-9-S1.zip › Additional File 1/Data1/MSData1/DS1MS21.png]

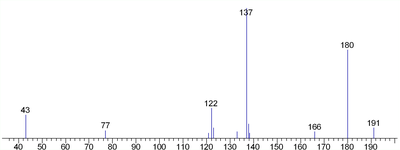

Supplement: Additional File 1 — Supporting interactive supplemental data for Figure 4, including machine readable MS data are given in Additional File 1.zip. To access these data, download this file and unzip the compressed archive, ensuring that the embedded directory structure is preserved. Once uncompressed, simply open IDS Figure 4.htm. JavaScript must be enabled in your web browser in order to fully access these files. These files will also be available on line via the Geochemical Transactions web site in the near future. [file 1467-4866-7-9-S1.zip › Additional File 1/Data1/MSData1/DS1MS22.png]

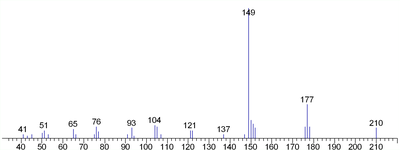

Supplement: Additional File 1 — Supporting interactive supplemental data for Figure 4, including machine readable MS data are given in Additional File 1.zip. To access these data, download this file and unzip the compressed archive, ensuring that the embedded directory structure is preserved. Once uncompressed, simply open IDS Figure 4.htm. JavaScript must be enabled in your web browser in order to fully access these files. These files will also be available on line via the Geochemical Transactions web site in the near future. [file 1467-4866-7-9-S1.zip › Additional File 1/Data1/MSData1/DS1MS23.png]

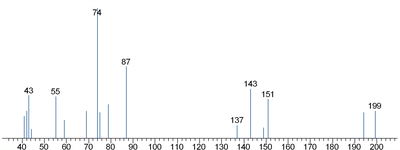

Supplement: Additional File 1 — Supporting interactive supplemental data for Figure 4, including machine readable MS data are given in Additional File 1.zip. To access these data, download this file and unzip the compressed archive, ensuring that the embedded directory structure is preserved. Once uncompressed, simply open IDS Figure 4.htm. JavaScript must be enabled in your web browser in order to fully access these files. These files will also be available on line via the Geochemical Transactions web site in the near future. [file 1467-4866-7-9-S1.zip › Additional File 1/Data1/MSData1/DS1MS24.png]

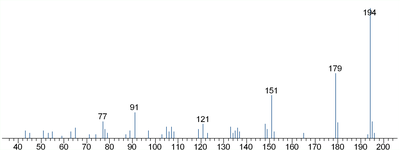

Supplement: Additional File 1 — Supporting interactive supplemental data for Figure 4, including machine readable MS data are given in Additional File 1.zip. To access these data, download this file and unzip the compressed archive, ensuring that the embedded directory structure is preserved. Once uncompressed, simply open IDS Figure 4.htm. JavaScript must be enabled in your web browser in order to fully access these files. These files will also be available on line via the Geochemical Transactions web site in the near future. [file 1467-4866-7-9-S1.zip › Additional File 1/Data1/MSData1/DS1MS25.png]

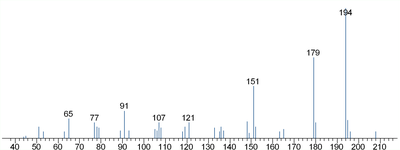

Supplement: Additional File 1 — Supporting interactive supplemental data for Figure 4, including machine readable MS data are given in Additional File 1.zip. To access these data, download this file and unzip the compressed archive, ensuring that the embedded directory structure is preserved. Once uncompressed, simply open IDS Figure 4.htm. JavaScript must be enabled in your web browser in order to fully access these files. These files will also be available on line via the Geochemical Transactions web site in the near future. [file 1467-4866-7-9-S1.zip › Additional File 1/Data1/MSData1/DS1MS26.png]

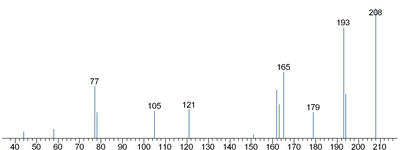

Supplement: Additional File 1 — Supporting interactive supplemental data for Figure 4, including machine readable MS data are given in Additional File 1.zip. To access these data, download this file and unzip the compressed archive, ensuring that the embedded directory structure is preserved. Once uncompressed, simply open IDS Figure 4.htm. JavaScript must be enabled in your web browser in order to fully access these files. These files will also be available on line via the Geochemical Transactions web site in the near future. [file 1467-4866-7-9-S1.zip › Additional File 1/Data1/MSData1/DS1MS27.png]

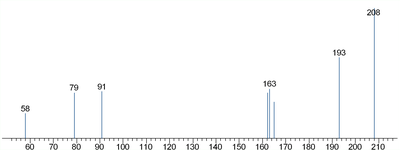

Supplement: Additional File 1 — Supporting interactive supplemental data for Figure 4, including machine readable MS data are given in Additional File 1.zip. To access these data, download this file and unzip the compressed archive, ensuring that the embedded directory structure is preserved. Once uncompressed, simply open IDS Figure 4.htm. JavaScript must be enabled in your web browser in order to fully access these files. These files will also be available on line via the Geochemical Transactions web site in the near future. [file 1467-4866-7-9-S1.zip › Additional File 1/Data1/MSData1/DS1MS28.png]

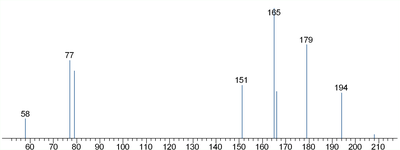

Supplement: Additional File 1 — Supporting interactive supplemental data for Figure 4, including machine readable MS data are given in Additional File 1.zip. To access these data, download this file and unzip the compressed archive, ensuring that the embedded directory structure is preserved. Once uncompressed, simply open IDS Figure 4.htm. JavaScript must be enabled in your web browser in order to fully access these files. These files will also be available on line via the Geochemical Transactions web site in the near future. [file 1467-4866-7-9-S1.zip › Additional File 1/Data1/MSData1/DS1MS29.png]

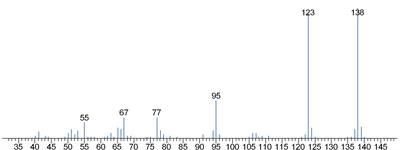

Supplement: Additional File 1 — Supporting interactive supplemental data for Figure 4, including machine readable MS data are given in Additional File 1.zip. To access these data, download this file and unzip the compressed archive, ensuring that the embedded directory structure is preserved. Once uncompressed, simply open IDS Figure 4.htm. JavaScript must be enabled in your web browser in order to fully access these files. These files will also be available on line via the Geochemical Transactions web site in the near future. [file 1467-4866-7-9-S1.zip › Additional File 1/Data1/MSData1/DS1MS3.png]

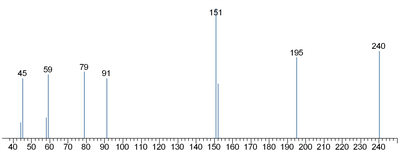

Supplement: Additional File 1 — Supporting interactive supplemental data for Figure 4, including machine readable MS data are given in Additional File 1.zip. To access these data, download this file and unzip the compressed archive, ensuring that the embedded directory structure is preserved. Once uncompressed, simply open IDS Figure 4.htm. JavaScript must be enabled in your web browser in order to fully access these files. These files will also be available on line via the Geochemical Transactions web site in the near future. [file 1467-4866-7-9-S1.zip › Additional File 1/Data1/MSData1/DS1MS30.png]

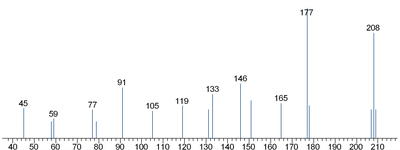

Supplement: Additional File 1 — Supporting interactive supplemental data for Figure 4, including machine readable MS data are given in Additional File 1.zip. To access these data, download this file and unzip the compressed archive, ensuring that the embedded directory structure is preserved. Once uncompressed, simply open IDS Figure 4.htm. JavaScript must be enabled in your web browser in order to fully access these files. These files will also be available on line via the Geochemical Transactions web site in the near future. [file 1467-4866-7-9-S1.zip › Additional File 1/Data1/MSData1/DS1MS31.png]

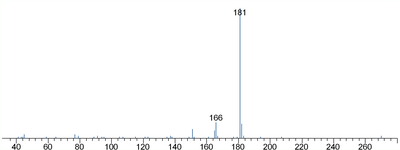

Supplement: Additional File 1 — Supporting interactive supplemental data for Figure 4, including machine readable MS data are given in Additional File 1.zip. To access these data, download this file and unzip the compressed archive, ensuring that the embedded directory structure is preserved. Once uncompressed, simply open IDS Figure 4.htm. JavaScript must be enabled in your web browser in order to fully access these files. These files will also be available on line via the Geochemical Transactions web site in the near future. [file 1467-4866-7-9-S1.zip › Additional File 1/Data1/MSData1/DS1MS32.png]

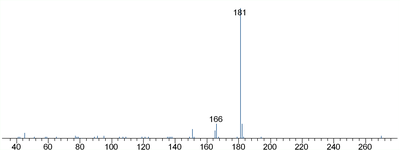

Supplement: Additional File 1 — Supporting interactive supplemental data for Figure 4, including machine readable MS data are given in Additional File 1.zip. To access these data, download this file and unzip the compressed archive, ensuring that the embedded directory structure is preserved. Once uncompressed, simply open IDS Figure 4.htm. JavaScript must be enabled in your web browser in order to fully access these files. These files will also be available on line via the Geochemical Transactions web site in the near future. [file 1467-4866-7-9-S1.zip › Additional File 1/Data1/MSData1/DS1MS33.png]
